# Self-Management Education Class Attendance and Health Care Provider Counseling for Physical Activity Among Adults with Arthritis — United States, 2019

**DOI:** 10.15585/mmwr.mm7042a2

**Published:** 2021-10-22

**Authors:** Lindsey M. Duca, Charles G. Helmick, Kamil E. Barbour, Dana Guglielmo, Louise B. Murphy, Michael A. Boring, Kristina A. Theis, Erica L. Odom, Yong Liu, Janet B. Croft

**Affiliations:** ^1^Epidemic Intelligence Service, CDC; ^2^Division of Population Health, National Center for Chronic Disease Prevention and Health Promotion, CDC; ^3^Oak Ridge Institute for Science and Education, Oak Ridge, Tennessee.

Arthritis is a highly prevalent and disabling condition among U.S. adults (*1*); arthritis-attributable functional limitations and severe joint pain affect many aspects of health and quality of life (*2*). Self-management education (self-management) and physical activity can reduce pain and improve the health status and quality of life of adults with arthritis; however, in 2014, only 11.4% and 61.0% of arthritis patients reported engaging in each, respectively. To assess self-reported self-management class attendance and health care provider physical activity counseling among adults with doctor-diagnosed arthritis, CDC analyzed 2019 Behavioral Risk Factor Surveillance System (BRFSS) data. In 2019, an age-standardized state median of one in six (16.2%) adults with arthritis reported ever attending a self-management class, and 69.3% reported ever receiving health care provider counselling to be physically active. Prevalences of both differed by state and sociodemographic characteristics; decreased with lower educational attainment, joint pain severity, and urbanicity; and were lower in men than in women. Health care providers can play an important role in promoting self-management class attendance and physical activity by counseling arthritis patients about their benefits and referring patients to evidence-based programs (*3*).

BRFSS is an annual, cross-sectional, state-based telephone survey conducted among the noninstitutionalized U.S. population aged ≥18 years.* In 2019, the median combined landline and cellular survey response rate for 49 states^†^ and the District of Columbia (DC) was 49.4% (range = 37.3%–73.1%).^§^ Participants were identified as having arthritis if they responded “yes” to the question, “Have you ever been told by a doctor or other health care professional that you have arthritis, rheumatoid arthritis, gout, lupus, or fibromyalgia?”^¶^ Among 135,862 adults with arthritis, self-management class attendance was defined by an affirmative response to the question, “Have you ever taken an educational course or class to teach you how to manage problems related to your arthritis or joint symptoms?” Respondents with arthritis were classified as having received health care provider counseling for physical activity if they answered “yes” to the question, “Has a doctor or other health professional ever suggested physical activity or exercise to help your arthritis or joint symptoms?”

Among adults with arthritis in 49 states and DC, state-specific unadjusted and age-standardized** prevalences (with 95% confidence intervals [CIs]) were calculated for self-management class attendance or having received health care provider counseling (counseling) to be physically active. Differences in the prevalences of these two outcomes by selected characteristics were assessed in age-adjusted^††^ logistic regression models that included age as a categorical covariate. All analyses accounted for BRFSS’s complex sampling design and sampling weights, based on iterative proportional fitting, were applied to make state-specific estimates representative of each state.^§§^ Analyses were conducted using SAS (version 9.4; SAS Institute) and SUDAAN (version 11.0; RTI International). This activity was reviewed by CDC and was conducted consistent with applicable federal law and CDC policy.^¶¶^

In 2019, among 49 states and DC, a median of 23.6% of respondents reported having arthritis. Among adults with arthritis, the median age-standardized prevalence of reported self-management class attendance was 16.2% (range = 9.8% [DC] to 24.9% [Hawaii]) (Table 1). Age-adjusted prevalence reflected lower self-management class attendance among men (15.4%) than among women (17.0%), among non-Hispanic White (15.6%) or Hispanic (17.0%) persons than among non-Hispanic Asian (20.9%), American Indian or Alaska Native (21.9%), or other or multiple race (21.2%) persons, and among those never married (15.0%) or a member of an unmarried couple (15.8%) than among those married (16.0%) or divorced, separated, or widowed (17.3%) (Table 2). Age-adjusted prevalence increased with higher educational attainment, urbanicity, federal poverty level, and joint pain severity. Groups with prevalences of self-management class attendance of <15.0% included persons with a high school education or less (12.8%); those employed (14.8%), unemployed (13.4%), or a student or homemaker (12.8%); those residing in micropolitan (14.5%) or rural areas (14.7%); those who were inactive in the last 30 days (12.9%); and those with no to mild joint pain (13.6%). No differences in prevalence by sexual orientation or body mass index were observed.

**TABLE 1:** Unadjusted and age-standardized* prevalence of self-management education class attendance**^†^** and receipt of health care provider counseling about physical activity**^§^** among adults with arthritis**^¶^** aged ≥18 years — Behavioral Risk Factor Surveillance System, United States,** 2019

Jurisdiction	Persons with arthritis			Self-management education class attendance			Health care provider physical activity counseling		
	Est. no.^††^	% (95% CI)		Est. no.^††^	% (95% CI)		Est. no.^††^	% (95% CI)	
		Unadjusted	Age-standardized		Unadjusted	Age-standardized		Unadjusted	Age-standardized
Median,^§§ ^%	NA	26.1	23.6	NA	15.7	16.2	NA	70.4	69.3
Alabama	1,273,000	33.9 (32.5–35.3)	30.4 (29.2–31.6)	191,000	15.1 (13.5–16.9)	17.3 (14.4–20.7)	871,000	69.0 (66.7–71.1)	69.1 (65.3–72.6)
Alaska	116,000	21.4 (19.4–23.5)	20.9 (19.2–22.8)	23,000	19.8 (15.7–24.6)	21.1 (14.7–29.2)	83,000	72.3 (68.0–76.2)	75.8 (69.8–80.9)
Arizona	1,301,000	23.6 (22.2–24.9)	21.0 (19.8–22.3)	226,000	17.5 (15.3–19.9)	16.2 (12.6–20.4)	907,000	70.1 (67.1–73.0)	67.1 (61.0–72.7)
Arkansas	715,000	31.2 (29.6–32.9)	28.5 (27.0–30.0)	104,000	14.6 (12.6–16.8)	14.7 (11.6–18.5)	466,000	66.3 (63.4–69.0)	63.2 (58.0–68.1)
California	6,007,000	19.8 (18.9–20.7)	18.4 (17.6–19.2)	1,192,000	19.9 (17.9–22.1)	20.4 (16.9–24.4)	336,000	72.7 (70.4–74.9)	70.3 (66.3–74.1)
Colorado	990,000	22.3 (21.4–23.2)	21.1 (20.2–22.0)	154,000	15.6 (14.0–17.4)	16.5 (13.8–19.7)	678,000	69.8 (67.7–71.9)	69.3 (65.5–72.8)
Connecticut	653,000	23.5 (22.5–24.6)	20.3 (19.4–21.3)	78,000	12.0 (10.6–13.6)	12.8 (9.8–16.5)	467,000	72.6 (70.3–74.7)	71.3 (66.2–75.9)
Delaware	208,000	27.4 (25.6–29.3)	23.6 (22.0–25.3)	33,000	15.6 (13.2–18.4)	15.4 (11.8–19.7)	152,000	73.1 (69.7–76.2)	69.1 (62.2–75.2)
District of Columbia	97,000	17.2 (15.7–18.9)	18.7 (17.3–20.3)	15,000	15.7 (12.9–18.9)	9.8 (7.4–12.7)	73,000	77.3 (72.7–81.3)	74.4 (66.0–81.3)
Florida	4,325,000	25.4 (24.1–26.7)	21.1 (20.0–22.3)	881,000	20.4 (17.9–23.2)	20.8 (16.4–26.2)	3,052,000	71.4 (68.9–73.7)	70.2 (65.4–74.5)
Georgia	1,902,000	23.8 (22.4–25.2)	22.2 (21.0–23.5)	301,000	15.9 (13.7–18.4)	17.0 (12.7–22.3)	1,260,000	67.0 (63.8–70.0)	63.4 (57.5–68.9)
Hawaii	230,000	20.9 (19.8–22.1)	18.4 (17.4–19.5)	48,000	20.8 (18.3–23.5)	24.9 (20.2–30.3)	159,000	69.7 (66.8–72.4)	66.7 (61.4–71.7)
Idaho	329,000	25.1 (23.4–26.8)	23.1 (21.5–24.7)	64,000	19.5 (16.4–23.1)	21.1 (15.4–28.3)	212,000	65.9 (62.3–69.4)	67.1 (61.0–72.6)
Illinois	2,409,000	24.7 (23.5–26.0)	22.5 (21.4–23.7)	415,000	17.2 (15.2–19.5)	15.8 (12.9–19.2)	1,715,000	71.6 (68.9–74.2)	70.5 (65.9–74.6)
Indiana	1,358,000	26.9 (25.9–28.0)	24.7 (23.7–25.7)	216,000	16.0 (14.4–17.7)	16.3 (13.5–19.5)	921,000	68.8 (66.6–70.9)	68.0 (64.2–71.6)
Iowa	618,000	25.7 (24.7–26.6)	23.0 (22.1–23.9)	94,000	15.4 (14.0–16.9)	17.0 (14.5–19.8)	408,000	67.3 (65.3–69.2)	65.5 (62.0–68.9)
Kansas	555,000	25.6 (24.7–26.5)	23.6 (22.7–24.4)	89,000	16.1 (14.6–17.6)	15.7 (13.3–18.4)	374,000	68.6 (66.6–70.5)	65.7 (62.1–69.1)
Kentucky	1,176,000	34.3 (32.7–35.9)	31.3 (29.8–32.9)	157,000	13.4 (11.5–15.4)	14.0 (11.3–17.0)	796,000	68.4 (65.8–70.9)	66.1 (61.9–70.0)
Louisiana	968,000	27.6 (26.1–29.2)	25.5 (24.2–26.9)	140,000	14.6 (12.5–16.8)	15.3 (12.2–18.9)	686,000	71.8 (69.0–74.5)	72.9 (68.5–76.9)
Maine	340,000	31.8 (30.5–33.1)	27.4 (26.1–28.8)	48,000	14.1 (12.6–15.7)	13.7 (11.2–16.8)	238,000	71.3 (69.0–73.4)	70.6 (66.0–74.8)
Maryland	1,107,000	23.9 (23.1–24.8)	21.6 (20.9–22.4)	178,000	16.2 (14.8–17.6)	17.7 (14.7–21.1)	826,000	75.3 (73.7–76.9)	75.2 (71.9–78.2)
Massachusetts	1,316,000	24.5 (23.3–25.7)	21.9 (20.8–23.0)	205,000	15.7 (13.9–17.7)	15.1 (12.3–18.4)	945,000	73.5 (71.1–75.8)	72.0 (67.6–76.0)
Michigan	2,373,000	30.8 (29.6–31.9)	27.2 (26.2–28.2)	345,000	14.6 (13.2–16.0)	14.5 (12.3–17.0)	1,665,000	71.0 (69.0–72.9)	70.6 (66.9–74.0)
Minnesota	928,000	21.7 (20.9–22.4)	19.4 (18.8–20.1)	175,000	19.0 (17.6–20.5)	18.4 (16.2–20.8)	629,000	69.1 (67.3–70.8)	67.5 (64.4–70.5)
Mississippi	650,000	28.8 (27.3–30.4)	26.3 (24.9–27.7)	92,000	14.2 (12.1–16.7)	18.5 (13.9–24.1)	442,000	68.7 (65.8–71.5)	69.5 (64.6–74.0)
Missouri	1,270,000	27.1 (25.8–28.4)	24.1 (22.9–25.2)	194,000	15.3 (13.6–17.3)	14.2 (11.6–17.3)	833,000	66.5 (63.8–69.0)	63.7 (58.9–68.3)
Montana	241,000	28.9 (27.7–30.2)	25.4 (24.3–26.6)	37,000	15.7 (13.9–17.6)	16.2 (13.3–19.5)	152,000	64.6 (62.1–67.0)	64.2 (60.0–68.2)
Nebraska	335,000	23.1 (22.3–24.0)	21.0 (20.2–21.7)	51,000	15.4 (14.0–16.9)	14.6 (12.2–17.4)	223,000	67.2 (65.3–69.1)	64.7 (60.9–68.4)
Nevada	531,000	22.7 (20.6–25.0)	20.7 (18.7–22.8)	96,000	18.2 (14.3–22.9)	15.4 (11.7–20.2)	366,000	69.0 (63.7–73.8)	70.2 (61.9–77.4)
New Hampshire	287,000	26.4 (25.0–27.9)	22.9 (21.5–24.2)	47,000	16.4 (14.5–18.6)	16.2 (12.5–20.6)	197,000	69.8 (67.0–72.6)	64.9 (58.7–70.7)
New Mexico	413,000	25.8 (24.4–27.3)	23.2 (21.9–24.5)	75,000	18.1 (15.8–20.6)	18.8 (15.1–23.2)	295,000	71.7 (68.9–74.3)	68.6 (63.7–73.1)
New York	3,302,000	22.1 (21.2–23.0)	19.9 (19.1–20.7)	472,000	14.4 (12.9–15.9)	12.8 (10.8–15.0)	2,357,000	72.1 (70.0–74.1)	69.6 (65.7–73.1)
North Carolina	2,172,000	27.0 (25.5–28.5)	24.4 (23.0–25.8)	412,000	19.0 (16.6–21.7)	21.5 (17.5–26.2)	607,000	74.5 (71.5–77.3)	75.0 (70.4–79.2)
North Dakota	147,000	25.4 (23.9–26.9)	24.2 (22.8–25.6)	18,000	12.6 (10.6–14.8)	12.6 (9.4–16.7)	93,000	64.6 (61.4–67.7)	59.9 (54.3–65.3)
Ohio	2,751,000	30.6 (29.5–31.8)	27.5 (26.4–28.6)	422,000	15.4 (13.9–17.1)	15.5 (13.2–18.2)	1,926,000	70.9 (68.8–72.8)	70.6 (67.0–73.9)
Oklahoma	790,000	27.0 (25.7–28.3)	25.0 (23.9–26.2)	128,000	16.3 (14.5–18.2)	16.7 (13.7–20.2)	522,000	67.1 (64.5–69.6)	65.0 (60.4–69.3)
Oregon	863,000	26.3 (25.0–27.6)	23.6 (22.5–24.8)	175,000	20.5 (18.3–22.8)	21.7 (18.5–25.2)	605,000	71.4 (68.7–74.0)	69.2 (65.1–72.9)
Pennsylvania	2,910,000	29.1 (27.7–30.5)	25.1 (24.0–26.3)	372,000	12.8 (11.2–14.7)	12.7 (10.0–15.9)	2,031,000	70.7 (68.2–73.1)	72.9 (68.8–76.6)
Rhode Island	224,000	26.8 (25.3–28.3)	23.8 (22.5–25.2)	33,000	14.9 (12.9–17.0)	15.3 (11.6–20.0)	168,000	75.7 (73.0–78.2)	75.5 (69.4–80.6)
South Carolina	1,114,000	28.2 (26.9–29.5)	25.0 (23.8–26.3)	172,000	15.5 (13.7–17.4)	13.6 (11.2–16.5)	760,000	68.8 (66.2–71.2)	64.7 (60.0–69.1)
South Dakota	176,000	26.7 (24.6–28.9)	24.1 (22.1–26.1)	32,000	18.0 (15.0–21.5)	18.1 (13.5–23.7)	120,000	69.2 (65.0–73.0)	70.2 (63.6–76.1)
Tennessee	1,598,000	30.6 (29.1–32.2)	28.0 (26.6–29.4)	241,000	15.2 (13.3–17.4)	16.2 (13.1–19.9)	1,071,000	67.9 (65.2–70.6)	66.5 (61.9–70.7)
Texas	4,398,000	20.7 (19.5–22.0)	20.1 (19.0–21.2)	602,000	13.9 (11.9–16.1)	13.9 (11.0–17.3)	3,125,000	72.0 (68.9–74.9)	69.4 (64.0–74.2)
Utah	519,000	23.1 (22.2–24.0)	24.0 (23.2–24.8)	85,000	16.5 (14.9–18.2)	17.6 (15.3–20.3)	366,000	71.7 (69.8–73.6)	71.2 (68.4–73.9)
Vermont	135,000	27.0 (25.6–28.6)	23.0 (21.7–24.4)	21,000	15.4 (13.4–17.5)	17.4 (13.3–22.6)	95,000	70.8 (67.9–73.6)	69.4 (63.2–75.0)
Virginia	1,730,000	26.3 (25.2–27.4)	24.0 (23.0–25.1)	286,000	16.6 (14.9–18.5)	17.7 (14.6–21.1)	1,206,000	70.7 (68.5–72.9)	71.6 (67.6–75.2)
Washington	1,439,000	24.6 (23.7–25.5)	22.5 (21.7–23.3)	248,000	17.3 (15.8–18.8)	17.0 (14.6–19.7)	1,007,000	70.8 (69.0–72.6)	71.5 (68.3–74.4)
West Virginia	585,000	41.4 (39.7–43.1)	36.4 (34.9–38.0)	73,000	12.4 (11.0–14.0)	12.1 (10.0–14.5)	383,000	66.1 (63.7–68.3)	65.4 (61.4–69.1)
Wisconsin	1,244,000	27.8 (26.3–29.3)	24.6 (23.3–26.0)	196,000	15.8 (13.7–18.1)	19.7 (15.3–25.0)	880,000	71.6 (68.8–74.2)	74.3 (69.6–78.5)
Wyoming	109,000	25.1 (23.5–26.8)	22.8 (21.3–24.3)	14,000	12.9 (10.8–15.3)	11.1 (8.3–14.7)	69,000	64.3 (60.8–67.7)	64.5 (58.0–70.5)
Guam	17,000	16.1 (14.0–18.5)	17.7 (15.6–20.0)	3,000	16.3 (12.5–21.0)	17.2 (12.2–23.6)	12,000	72.7 (64.3–79.8)	66.8 (57.0–75.3)
Puerto Rico	574,000	21.2 (20.0–22.4)	18.4 (17.4–19.4)	48,000	8.3 (6.8–10.2)	11.4 (7.8–16.4)	412,000	72.5 (69.5–75.3)	73.2 (67.5–78.2)

**Abbreviations:** CI = confidence interval; Est. = estimated; NA = not applicable.

* Estimates were age-standardized to the 2000 Projected U.S. Population aged ≥18 years using three age groups: 18−44, 45–64, and ≥65 years. https://www.cdc.gov/nchs/data/statnt/statnt20.pdf

^†^ Respondents were classified as attending a self-management education course if they answered “yes” to the question, “Have you ever taken an education course or class to teach you how to manage problems related to your arthritis or joint symptoms?”

^§^ Respondents were classified as receiving health care provider counseling to be physically active if they answered “yes” to the question, “Has a doctor or other health professional ever suggested physical activity or exercise to help your arthritis or joint symptoms?”

^¶^ Respondents were classified as having arthritis if they responded “yes” to the question, “Have you ever been told by a doctor or other health care professional that you have arthritis, rheumatoid arthritis, gout, lupus, or fibromyalgia?”

** In 2019, New Jersey did not collect enough data to meet the minimum requirement for inclusion in the Behavioral Risk Factor Surveillance System public-use data set.

^††^ Estimated number represents the weighted estimated number of adults with arthritis who reported the outcome of interest (e.g., health care provider counseling to be physically active and self-management education class attendance) rounded to the nearest thousand.

^§§^ Median calculated for 49 states and the District of Columbia.

**TABLE 2:** Overall, age-adjusted, and age-specific* prevalence of self-management education class attendance**^†^** and receipt of health care provider counseling for physical activity**^§^** among adults with arthritis aged ≥18 years,**^¶^** by selected characteristics — Behavioral Risk Factor Surveillance System, United States,** 2019

Characteristic	Unweighted sample size	% (95% CI)	
		Self-management education class attendance	Health care provider counseling for physical activity
**Overall (unadjusted)**	135,862	16.4 (15.9–16.8)	70.8 (70.3–71.2)
**Overall (age-adjusted)**	135,862	16.3 (15.9–16.7)	70.8 (70.3–71.3)
**Age-specific estimates**			
**Age group, yrs^††^**			
18–44	11,665	16.9 (15.7–18.1)	67.9 (66.4–69.4)
45–64	47,991	16.4 (15.8–17.1)	71.2 (70.4–71.9)
≥65	76,206	16.1 (15.5–16.7)	71.4 (70.8–72.1)
**Age-adjusted estimates**			
**Sex**			
Female	83,885	17.0 (16.5–17.6)	74.5 (73.9–75.1)
Male	51,977	15.4 (14.7–16.0)	65.3 (64.4–66.1)
**Race/Ethnicity**			
White, NH	112,595	15.6 (15.2–16.0)	69.2 (68.7–69.7)
Black, NH	10,407	18.1 (16.8–19.5)	76.0 (74.5–77.5)
Hispanic	5,317	17.0 (15.0–19.2)	75.3 (72.9–77.5)
Asian, NH	1,174	20.9 (15.7–27.2)	75.1 (69.5–80.0)
American Indian or Alaska Native, NH	2,323	21.9 (17.7–26.8)	67.8 (63.2–72.0)
Other or multiple race, NH	4,046	21.1 (18.7–23.7)	72.6 (69.9–75.1)
**Marital status**			
Married	67,122	16.0 (15.5–16.6)	70.7 (70.0–71.4)
Divorced, separated, or widowed	52,525	17.3 (16.6–18.1)	70.4 (69.6–71.2)
Never married	12,615	15.0 (13.7–16.5)	71.7 (70.1–73.3)
Member of an unmarried couple	2,906	15.8 (13.4–18.6)	71.0 (67.9–73.8)
**Highest level of education**			
Less than high school graduate	10,894	12.8 (11.5–14.1)	67.2 (65.6–68.8)
High school graduate or equivalent	39,281	12.8 (12.1–13.5)	69.2 (68.3–70.1)
Technical school or some college	40,588	19.2 (18.4–20.0)	72.4 (71.5–73.2)
College degree or higher	44,763	18.9 (18.2–19.7)	72.6 (71.7–73.4)
**Employment status**			
Employed or self-employed	42,601	14.8 (14.1–15.5)	67.7 (66.8–68.6)
Unemployed	4,487	13.4 (11.5–15.4)	69.6 (67.0–72.1)
Retired	62,828	17.6 (16.7–18.5)	72.6 (71.6–73.5)
Unable to work or disabled	18,080	19.3 (18.2–20.5)	73.6 (72.4–74.8)
Other (student or homemaker)	6,533	12.8 (11.4–14.3)	72.6 (70.4–74.7)
**Federal poverty level^§§^**			
≤125% FPL	21,802	16.1 (15.1–17.2)	71.8 (70.6–73.0)
>125% to ≤200% FPL	21,593	15.9 (14.9–17.0)	70.7 (69.5–71.9)
>200% to ≤400% FPL	32,007	16.4 (15.6–17.2)	70.9 (69.9–71.9)
>400% FPL	34,014	17.1 (16.2–17.9)	70.6 (69.6–71.6)
**Urban-rural status** ^¶¶^			
Large central metro	16,929	17.8 (16.7–18.9)	73.6 (72.3–74.9)
Large fringe metro	23,940	16.1 (15.2–16.9)	71.4 (70.3–72.4)
Medium metro	28,118	16.6 (15.9–17.4)	71.0 (70.1–71.9)
Small metro	19,627	16.2 (15.2–17.1)	68.4 (67.2–69.6)
Micropolitan	23,087	14.5 (13.7–15.4)	68.1 (66.9–69.2)
Rural (non-core)	24,161	14.7 (13.8–15.6)	66.0 (64.7–67.3)
Sexual orientation***			
Straight	73,022	15.9 (15.3–16.4)	71.1 (70.4–71.8)
Lesbian, gay, bisexual, queer, or questioning	4,264	15.5 (13.0–18.5)	72.3 (69.5–75.0)
**Engaged in physical activity in past month^†††^**			
Yes	87,299	18.0 (17.5–18.6)	73.1 (72.5–73.7)
No	42,960	12.9 (12.3–13.6)	66.5 (65.6–67.4)
**Body mass index (kg/m^2^)**			
Underweight or healthy weight (<25)	32,173	16.4 (15.5–17.3)	66.9 (65.8–67.9)
Overweight (25 to <30)	43,153	16.2 (15.5–17.0)	69.4 (68.5–70.3)
Obesity (≥30)	50,837	16.5 (15.8–17.2)	74.5 (73.7–75.2)
**Joint pain severity^§§§^**			
None/Mild	62,913	13.6 (13.0–14.2)	66.3 (65.5–67.0)
Moderate	32,184	17.8 (16.9–18.7)	74.7 (73.7–75.7)
Severe	38,465	19.1 (18.3–19.9)	74.5 (73.6–75.3)

**Abbreviations:** CI = confidence interval; FPL = federal poverty level; NH = non-Hispanic.

* Except for the age groups category and the unadjusted overall variables, age-adjusted estimates were generated in weighted logistic regression models that included age as a categorical covariate using the following cut points: 18−44, 45−64, and ≥65 years.

^†^ Respondents were classified as attending a self-management education course if they responded “yes” to the question, “Have you ever taken an education course or class to teach you how to manage problems related to your arthritis or joint symptoms?”

^§^ Respondents were classified as receiving health care provider counseling to be physically active if they responded “yes” to the question, “Has a doctor or other health professional ever suggested physical activity or exercise to help your arthritis or joint symptoms?”

^¶^ Respondents were classified as having arthritis if they responded “yes” to the question, “Have you ever been told by a doctor or other health care professional that you have arthritis, rheumatoid arthritis, gout, lupus, or fibromyalgia?”

** In 2019, New Jersey did not collect sufficient data to meet the minimum requirement for inclusion in the Behavioral Risk Factor Surveillance System public-use data set.

^††^ Age-specific estimates.

^§§^ Federal poverty level is the ratio of total family income to federal poverty guideline per family size.

^¶¶^ Urban-rural status was categorized using the National Center for Health Statistics 2013 Urban-Rural Classification Scheme for Counties. https://www.cdc.gov/nchs/data/series/sr_02/sr02_166.pdf

*** Sexual orientation was asked in 30 states (Alaska, Arizona, Colorado, Connecticut, Delaware, Florida, Georgia, Hawaii, Idaho, Iowa, Kansas, Louisiana, Maryland, Minnesota, Mississippi, Montana, New York, North Carolina, Ohio, Oklahoma, Rhode Island, South Carolina, Tennessee, Texas, Utah, Vermont, Virginia, Washington, West Virginia, and Wisconsin).

^†††^ Physical activity was defined using the question, “During the past month, other than your regular job, did you participate in any physical activities or exercises such as running, calisthenics, golf, gardening, or walking for exercise?”

^§§§^ For the question, “On a scale of 0 to 10 where 0 is no pain or aching and 10 is pain or aching as bad as it can be, during the past 30 days, how bad was your joint pain on average,” an answer of 0−4 was defined as none or mild, an answer of 5−6 was defined as moderate, and an answer of 7−10 was defined as severe.

Among adults with arthritis who reported having received counseling to be physically active, the median age-standardized prevalence was 69.3% (range = 59.9% [North Dakota] to 75.8% [Alaska]) (Table 1). The age-specific percentage of adults with arthritis who reported receipt of counseling was lowest among those aged 18–44 years (Table 2). Age-adjusted reporting of receipt of counseling was less prevalent among those physically inactive (66.5%) in the last 30 days than among those active (73.1%), among non-Hispanic American Indian or Alaska Native (67.8%) or non-Hispanic White (69.2%) persons than among Hispanic (75.3%), or non-Hispanic Asian or Black persons (75.1% and 76.0%, respectively), and among those employed (67.7%) or unemployed (69.6%) than among those who were retired (72.6%) or unable to work or disabled (73.6%). Prevalence of receiving counseling increased with increasing education, urbanicity, body mass index, and joint pain severity. Groups among which <67.0% had received counseling were men (65.3%), those residing in rural areas (66.0%), those who were inactive in the last 30 days (66.5%), those who were underweight or healthy weight (66.9%), and those who had no to mild joint pain (66.3%). Prevalence of receiving physical activity counseling was similar across federal poverty level, marital status, and sexual orientation categories. No clear regional patterns in the unadjusted and age-standardized prevalence of either self-management class attendance or counseling to be physically active were noted.

## Discussion

The prevalence of self-management class attendance and receipt of health care provider counseling to be physically active among adults with arthritis varied considerably across states and by participant characteristics, with no clear regional patterns. Among adults with arthritis, self-management class attendance was low among all persons. The specific groups identified with low self-management class attendance and receipt of physical activity counseling were men, persons with a high school education or less, and those residing in small cities or rural areas. Opportunities for increasing health care provider counseling and interventions focused on improving self-management class attendance and physical activity among persons living with arthritis should continue for all, but especially for those groups with lower engagement in these activities.

The benefits of self-management courses and counseling to engage in physical activity are well established health goals for the nation, each of which was codified and evaluated in Healthy People 2020. The relevant Healthy People 2020 arthritis objective target*** of 11.7% of persons with arthritis attending self-management classes indicated slow progress and was almost attained in 2014 (11.4%) as reported in the National Health Interview Survey (NHIS) (*4*). Similarly, advancement toward the Healthy People 2020 arthritis objective target^†††^ of 57.4% of adults with arthritis receiving physical activity counseling indicated good progress and was surpassed in the 2014 NHIS, when 61.0% of adults with arthritis reported receiving such counseling (*5*).

Among the known benefits of physical activity for adults with arthritis are improved mood, strength, and endurance and reduced arthritis-related joint pain, stiffness, and fatigue (*6*). Multiple professional organizations recommend that health care providers counsel adults with arthritis to engage in physical activity (*7*); however, a barrier commonly reported by providers is having insufficient training to counsel patients with arthritis (*8*). Health care providers can counsel patients about safely increasing physical activity using evidence-based, arthritis-appropriate, physical activity programs^§§§^ available in communities across the country. These include low-impact group aquatic exercise (e.g., Arthritis Foundation Aquatic Program); EnhanceFitness, which incorporates balance activities; Fit and Strong!, which emphasizes flexibility, strength training, aerobic walking and health education to promote behavior change; and Walk with Ease, which combines self-paced walks with instruction on health-related topics and can be delivered as a group or self-directed activity, both of which accommodate physical distancing, as recommended during the COVID-19 pandemic.

Recommending self-management class attendance while counseling persons with arthritis to engage in physical activity might be the most effective strategy for increasing physical activity. A health care provider’s recommendation to attend a self-management workshop is strongly associated with self-management class attendance (*9*). A meta-analysis of health outcomes, health behaviors, and health care utilization related to self-management programs found that persons with arthritis who received a health care provider recommendation to attend a self-management class were nine times more likely to attend a class than were those who did not receive a recommendation (*10*). The analysis found that aerobic physical activity increased after attendance in the generic, evidence-based self-management course^¶¶¶^ (Chronic Disease Self-Management Program [CDSMP]) and persisted for 1 year after attending the class (*10*). CDSMP is a workshop tailored to adults with chronic conditions (including arthritis) and other comorbidities which are also common among adults with arthritis (*1*); the workshop teaches improved self-efficacy and skills, resulting in better arthritis outcomes. Benefits of CDSMP include improved health status (e.g., reduced pain, and improved function and psychological health), improved health behaviors (e.g., increased physical activity, and improved healthful eating, pain-coping strategies, and medication adherence), and improved communication with health care providers. CDSMP is offered in a Spanish-language version (Tomando Control de su Salud) and virtually by the Better Choices, Better Health program.

The findings in this report are subject to at least three limitations. First, BRFSS data rely on self-report and might be subject to recall, social desirability, and other biases. Second, low response rates that differ by state might bias study findings; however, the weighting methodology accounts for nonresponse. Finally, the question to ascertain self-management class attendance did not establish whether respondents attended an evidence-based self-management course. A strength of this study is the use of recent data with a large sample size that allowed analyses of detailed characteristics in 49 states, DC, and two U.S. territories. In addition, the prevalence estimates generated are representative at the state level.

Self-management class attendance and health care provider counseling for physical activity varied by state and sociodemographic characteristics among adults with arthritis. Public health professionals and medical groups can help improve patient self-management behaviors and outcomes among patients with arthritis by equipping health care providers**** with the tools and information they need to counsel adults with arthritis to be active and recommend evidence-based physical activity and self-management programs.

**Summary**

**What is already known about this topic?**

Arthritis is a prevalent chronic condition. Self-management education and health care provider counseling encouraging engagement in physical activity can improve the health of adults with arthritis; however, in 2014, only 11.4% and 61.0% of arthritis patients reported engaging in each, respectively.

**What is added by this report?**

In 2019, a median of 16.2% adults with arthritis attended a self-management class, and 69.3% received provider counseling for physical activity. Prevalences differed by state and sociodemographic characteristics.

**What are the implications for public health practice?**

Equipping health care providers with the tools to counsel arthritis patients about the benefits of physical activity and self-management education and support referrals to evidence-based programs is needed to improve adoption of these behaviors.

## Footnotes

*
https://www.cdc.gov/brfss/about/index.htm

† In 2019, New Jersey did not collect sufficient data to meet the minimum requirement for inclusion in the BRFSS public-use data set.

§
https://www.cdc.gov/brfss/annual_data/2019/pdf/2019-response-rates-table-508.pdf

¶
https://www.cdc.gov/arthritis/basics/types.html

** Estimates were age-standardized to the 2000 U.S. Projected Population aged ≥18 years using three age groups: 18−44, 45–64, and ≥65 years to allow for state-to-state comparisons. https://www.cdc.gov/nchs/data/statnt/statnt20.pdf

†† Age-adjusted estimates were generated in weighted logistic regression models that included age as a categorical covariate with the following cut points: 18−44 years, 45–64 years, and ≥65 years.

§§
http://citeseerx.ist.psu.edu/viewdoc/download?doi=10.1.1.684.5837&rep=rep1&type=pdf

¶¶ 45 C.F.R. part 46.102(l)(2), 21 C.F.R. part 56; 42 U.S.C. Sect. 241(d); Sect. U.S.C. Sect. 552a; 44 U.S.C. Sect. 3501 et seq.

*** Healthy People 2020 self-management education objective AOCBC-8, “Increase the proportion of adults with doctor-diagnosed arthritis who have had effective, evidence-based arthritis education as an integral part of the management of their condition.” https://www.healthypeople.gov/2020/topics-objectives/topic/Arthritis-Osteoporosis-and-Chronic-Back-Conditions/objectives

††† Healthy People 2020 health care provider counseling for physical activity objective AOCBC-7.2, “Increase the proportion of adults with doctor-diagnosed arthritis who receive health care provider counseling for physical activity or exercise.” https://www.healthypeople.gov/2020/topics-objectives/topic/Arthritis-Osteoporosis-and-Chronic-Back-Conditions/objectives

§§§
https://www.cdc.gov/arthritis/interventions/physical-activity.html

¶¶¶
https://www.cdc.gov/arthritis/interventions/self_manage.htm

****
https://www.cdc.gov/arthritis/healthcare/index.html
